# Ten rules for robust taxonomy in ecological datasets

**DOI:** 10.1111/nph.71658

**Published:** 2026-10-12

**Authors:** Renato A. Ferreira de Lima, Guilherme S. Grittz, Moabe F. Fernandes, Hans ter Steege, Kyle G. Dexter, Oliver Phillips, Marli P. Morim, Jérôme Chave, Géraldine Derroire, Filipe M. França, Tamara Heartsill‐Scalley, Ricardo A. Segovia, Caroline E. R. Lehmann, Luis Cayuela, Karina Banda, Cecilia Blundo, Jesús Aguirre‐Gutiérrez, André L. de Gasper, R. Toby Pennington

**Affiliations:** ^1^ Departamento de Ciências Biológicas, Escola Superior de Agricultura “Luiz de Queiroz” Universidade de São Paulo Avenida Pádua Dias, 11 13418‐900 Piracicaba Brazil; ^2^ Programa de Pós‐Graduação em Recursos Florestais, Escola Superior de Agricultura “Luiz de Queiroz” Universidade de São Paulo Avenida Pádua Dias, 11 13418‐900 Piracicaba Brazil; ^3^ Royal Botanic Gardens Kew Richmond TW9 3AB Surrey UK; ^4^ Naturalis Biodiversity Center 2333 CR Leiden the Netherlands; ^5^ Quantitative Biodiversity Dynamics, Department of Biology Utrecht University 3584 CH Utrecht the Netherlands; ^6^ Dipartimento di Scienze della Vita e Biologia dei Sistemi Università di Torino 10123 Turin Italy; ^7^ School of GeoSciences University of Edinburgh EH8 9XP Edinburgh UK; ^8^ Taxonomy and Macroecology, Royal Botanic Garden Edinburgh EH3 5LR Edinburgh UK; ^9^ School of Geography University of Leeds LS2 9JT Leeds UK; ^10^ Jardim Botânico do Rio de Janeiro Rua Pacheco Leão, 915 22460‐030 Rio de Janeiro Brazil; ^11^ Centre de Recherche Biodiversité Environnement UMR 5300 (CNRS/IRD/UPS/INPT) 31062 Toulouse Cedex 9 France; ^12^ Cirad, UPR Forêts et Sociétés University of Montpellier 34398 Montpellier France; ^13^ Department of Forestry University of Brasilia Campus Darcy Ribeiro 70910‐900 Brasília Brazil; ^14^ School of Biological Sciences University of Bristol BS8 1TQ Bristol UK; ^15^ Programa de Pós‐Graduação em Ecologia Universidade Federal do Pará 66075‐110 Belém Brazil; ^16^ International Institute of Tropical Forestry, USDA Forest Service 00926‐1119 Río Piedras Puerto Rico; ^17^ Departamento de Botánica Universidad de Concepción 4070386 Concepción Chile; ^18^ Instituto de Ecología y Biodiversidad (IEB) 7800024 Santiago Chile; ^19^ Department of Biology, ESCET Universidad Rey Juan Carlos 28933 Madrid Spain; ^20^ Fundación Ecosistemas Secos de Colombia 081001 Puerto Colombia Colombia; ^21^ Instituto de Ecología Regional Universidad Nacional de Tucumán – Consejo Nacional de Investigaciones Científicas y Técnicas (CONICET) Casilla de Correo 34 4107 Tucumán Argentina; ^22^ Environmental Change Institute University of Oxford OX1 3QY Oxford UK; ^23^ Facultad de Ciencias Forestales y Ambientales Universidad Juárez del Estado de Durango 34120 Durango Mexico; ^24^ Departamento de Ciências Naturais Universidade Regional de Blumenau 89030‐903 Blumenau Brazil; ^25^ Department of Biology Washington University St Louis MO 63130 USA; ^26^ Missouri Botanical Garden St Louis MO 63130 USA; ^27^ Geography Department, College of Life and Environmental Sciences University of Exeter EX4 4QE Exeter UK

**Keywords:** biodiversity, data curation and exchange, taxonomic backbones, taxonomic standardization, voucher specimens

## Abstract

Accurate taxonomy, including species identification and naming, underpins ecological inference, yet taxonomic information in ecological datasets is often inconsistently recorded or weakly standardized. With the increasing integration of ecological datasets across taxa and regions, datasets with poor taxonomic practices can hinder data exchange and propagate errors. We propose 10 rules to improve the storage and management of taxonomic information in ecological datasets. These rules draw on our experiences in plant ecology and taxonomy, but the conclusions may be generalized to other organisms and disciplines. They emphasize aspects from species identification to the permanent storage of verifiable records (e.g. voucher specimens) and include taxonomic standardization, nomenclature, validation, and updating. We also address the treatment of morphotypes and incomplete identifications, the use of automated name‐matching tools, and (meta)data standards. Closer collaboration between ecologists and taxonomists is key to ensuring more robust datasets. The application of these rules consumes more time and resources, but they enhance data quality and interoperability, saving time during data integration and improving the reliability and scalability of ecological research.

## Graphical abstract

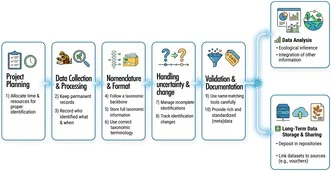

## Resumo

Uma taxonomia precisa, incluindo a identificação e nomenclatura das espécies, é fundamental para a inferência em ecologia. Entretanto, as informações taxonômicas em conjuntos de dados ecológicos frequentemente são pouco padronizadas. Com a integração contínua de dados ecológicos entre diferentes grupos taxonômicos e regiões, a adoção de práticas inadequadas pode dificultar o intercâmbio de dados e propagar erros. Neste trabalho, nós propomos dez regras para aprimorar o armazenamento e curadoria de informações taxonômicas em conjuntos de dados ecológicos. Essas regras foram baseadas em nossa expertise em ecologia e taxonomia vegetal, mas suas conclusões podem ser generalizadas para outros organismos e disciplinas. As regras enfatizam aspectos que vão desde a identificação de espécies até o armazenamento permanente de registros verificáveis (por exemplo, espécimes‐voucher) e incluem assuntos referentes a nomenclatura, padronização, validação e atualização taxonômica. Também abordamos o tratamento de morfotipos e identificações incompletas, o uso de ferramentas automatizadas de correspondência de nomes e padrões de (meta)dados. Uma colaboração mais estreita entre ecólogos e taxonomistas é essencial para garantir conjuntos de dados mais robustos. A aplicação dessas regras demanda mais tempo e recursos, mas melhora a qualidade dos dados e a interoperabilidade, economizando tempo durante a integração de dados e aprimorando a confiabilidade e escalabilidade em ecologia.

## Resumen

Una taxonomía precisa, que incluya la identificación y asignación de nombres científicos, es fundamental para la inferencia ecológica; sin embargo, la información taxonómica suele estar poco estandarizada en las bases de datos ecológicos. Con el aumento en la integración de bases de datos ecológicos de distintas taxones y regiones, los conjuntos de datos con prácticas taxonómicas precarias pueden propagar errores y dificultar el intercambio de datos. En este manuscrito, proponemos diez reglas para mejorar el almacenamiento y el manejo de información taxonómica en bases de datos ecológicos. Estas reglas se basan en nuestra experiencia en ecología y taxonomía de plantas, pero pueden extrapolarse a otros organismos y disciplinas. Estas reglas enfatizan aspectos desde la identificación de especies hasta el almacenamiento permanente de registros verificables (e.g. especímenes de colecciones), también incluyen estandarización taxonómica, nomenclatura, validación y actualizaciones. De igual forma, se tiene en cuenta el manejo de morfotipos e identificaciones incompletas, más el uso de herramientas automatizadas para la revisión de nombres y metadatos. Proponemos que una relación más cercana entre ecólogos y taxónomos resulta clave para asegurar conjuntos de datos más sólidos y confiables. La aplicación de estas reglas requiere de tiempo y recursos, pero al usarlas se mejora la calidad de los datos y su interoperabilidad, ahorrando tiempo durante la integración de datos y mejorando la confiabilidad y escalabilidad de estudios ecológicos.

## Résumé

Une taxonomie précise, comprenant l’identification et la dénomination des espèces, est à la base des déductions écologiques; or, les informations taxonomiques contenues dans les données écologiques sont souvent mal normalisées. Avec l’intégration croissante des données écologiques entre les taxons et les régions, les données fondées sur des pratiques taxonomiques insuffisantes peuvent entraver le partage de données et propager des erreurs. Nous proposons dix règles visant à améliorer le stockage et la gestion des informations taxonomiques dans les données écologiques. Ces règles s’appuient sur notre expérience en écologie végétale et en taxonomie, mais les conclusions peuvent être généralisées à d’autres organismes et disciplines. Elles mettent l’accent sur l’identification des espèces et le stockage permanent d’enregistrements vérifiables (par exemple, les spécimens de référence), et incluent la normalisation taxonomique, la nomenclature, la validation et la mise à jour des données. Nous abordons également la gestion des morphotypes et des identifications incomplètes, l’utilisation d’outils automatisés de mise en correspondance des noms, ainsi que les métadonnées. Une collaboration plus étroite entre écologistes et taxonomistes est essentielle pour garantir la qualité des jeux de données. L’application de ces règles nécessite davantage de temps et de ressources, mais elle améliore la qualité et l’interopérabilité des données, ce qui permet de gagner du temps lors de l’intégration des données et d’améliorer la fiabilité et le passage à l’échelle en écologie.

## Introduction

Ecological datasets store information ranging from species (e.g. abundance, demography, traits, and interactions) to ecosystem levels (e.g. productivity and biomass stocks). With the increasing availability and quantity of information, ecological datasets have become invaluable resources for understanding life on Earth (Heberling *et al*., 2021). Critical information in any ecological dataset includes the names of taxa, especially at the species level. This information is useful for documenting species distributions, their changes in space and time, and for understanding the factors driving these changes (Bottin *et al*., 2020; Alba *et al*., 2021). Ecology also relies upon species identifications, and species names provide access to information, including evolutionary relationships, functional traits, conservation status, sociocultural value, and economic uses (Patterson *et al*., 2010). Therefore, accurate species identification and naming are fundamental to ecological research (Sandall *et al*., 2023).

Accurate species identification is often challenging. The underlying taxonomy and systematics on which identification relies are dynamic. Earth has millions of species, and even for flowering plants, which dominate most ecosystems, thousands of new species are described each year (Cheek *et al*., 2020). Many clades remain poorly studied, particularly in tropical regions, making precise identification challenging and sometimes impossible (Li *et al*., 2025). Even for well‐known clades with recently revised taxonomy, species identification can still be difficult due to subtle differentiation in morphology, lack of complete evidence for identification (e.g. flowers or fruits for plants), the lack of taxonomic resources (e.g. identification keys/guides, taxonomists, or a combination of these), or the logistical challenges in collecting samples (Dexter *et al*., 2010; Gomes *et al*., 2013; Engel *et al*., 2021). Moreover, accepted names reflect our current understanding of species boundaries, so it is common for taxonomic names to change as new evidence becomes available (e.g. additional specimens or new information such as chemistry and DNA data). Taking all these issues into account can help determine the taxonomic quality of a dataset and the extent to which it supports or undermines ecological inferences (Bortolus, 2008; Stropp *et al*., 2022; Augustine *et al*., 2024).

Although the challenges related to species delimitation and identification cannot be easily solved, the standardization of taxonomic information is more readily achieved (Sandall *et al*., 2023). Ecological datasets, however, have adopted standards more recently and less frequently than datasets in other biological fields (Michener, 2015; Roche *et al*., 2015), such as biological collections that widely adopt the Darwin Core standard (Wieczorek *et al*., 2012). There are well‐established ecological examples (e.g. the Long‐Term Ecological Research and the Terrestrial Ecosystem Research Network) that have successfully adopted data standards, but these examples remain rare. The lack of consistent taxonomic standardization poses significant challenges for ecological data managers and users (Kervin *et al*., 2013; Feng *et al*., 2022), particularly when integrating multiple datasets. It also hinders the use of ecological datasets for other purposes and fields, including the discovery of new species (Baker *et al*., 2017).

Here, we provide 10 rules for storing and managing taxonomic information in ecological datasets. These rules are presented in a way that can guide researchers in creating or maintaining databases (Fig. 1). They are primarily designed for datasets that store information on plant species sampled one or more times in observational or experimental settings (e.g. vegetation inventories). They may be generalizable to other organisms (e.g. animals, fungi) or to any datasets in which species identification is required (e.g. fossils, pollen, sound recordings, DNA sequences).

**Fig. 1:**
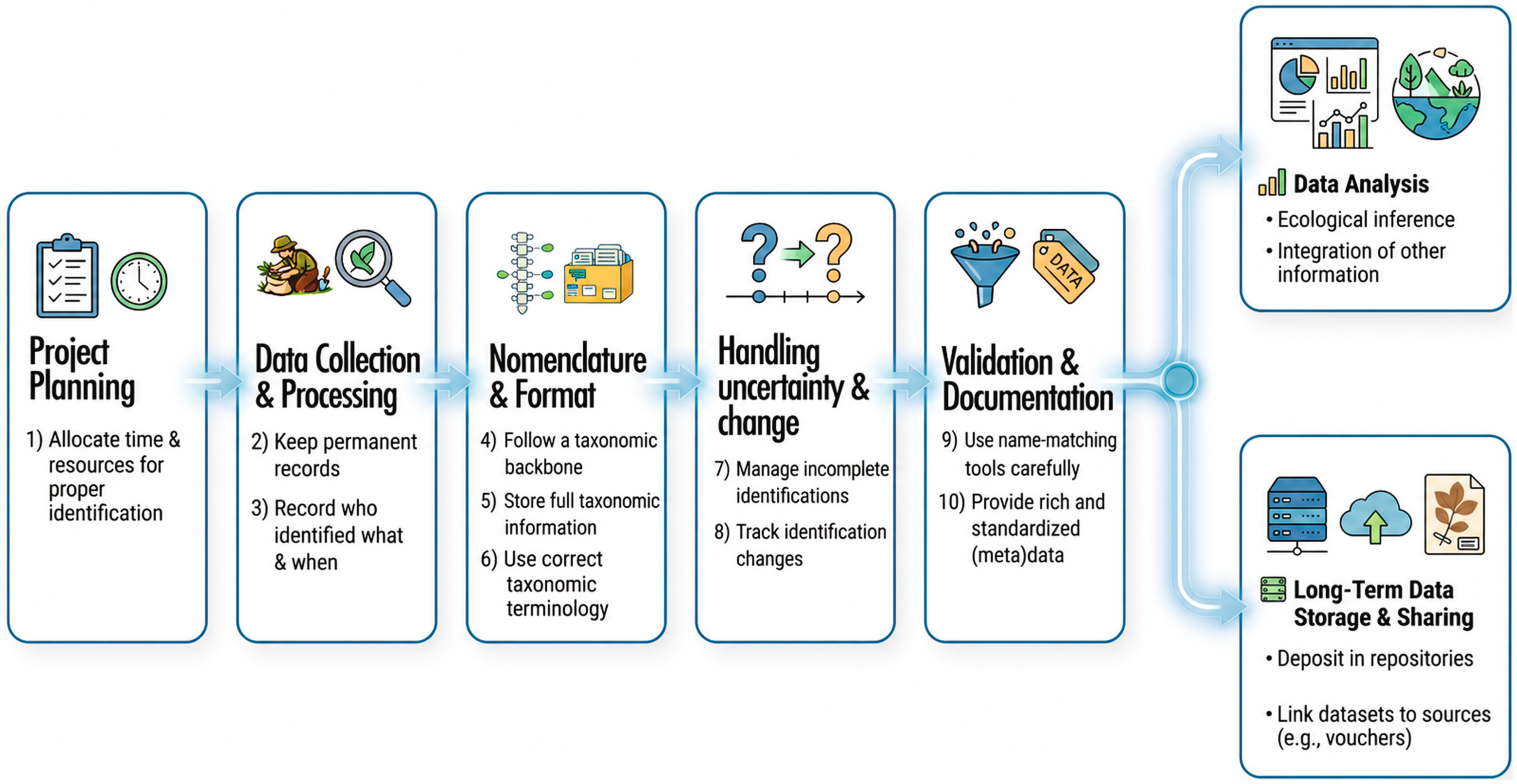
A workflow for better use and integration of taxonomic‐related information into ecological datasets. This schematic represents the 10 rules detailed in the text, organized for researchers and database managers to use as a practical guide. Rules were grouped by subject, and the workflow concludes with examples of possible uses that would benefit from more robust, well‐documented taxonomic information from the outset.

## Rule 1: Identifying species requires planning and resources

The taxonomic resolution required in an ecological study depends on its questions. While not all studies require species‐level identification (e.g. quantifying vegetation structure or growth), there are significant benefits in achieving this (Bevilacqua *et al*., 2021), as outlined in the introduction. However, taxonomic identification to the species level is laborious, beginning with planning the protocols, materials, equipment, and permits required to collect, identify, process, store, and transport voucher specimens. Establishing early collaboration with taxonomists and using taxonomic keys and descriptions are crucial but time‐consuming steps (Baker *et al*., 2017). Moreover, since collecting vouchers for every individual is rarely feasible (see Rule 2), taxonomic robustness depends on how consistently morphotypes (see Rule 7 and Box 1) are recognized and recorded in the field (Krell, 2004). Inconsistent or erroneous field morphotyping is a common and often overlooked source of error in ecological datasets (Morrison, 2016). Setting clear protocols, providing training for the consistent recognition and labeling of morphotypes across collectors and, whenever feasible, including taxonomists in field teams are therefore important elements of project planning (Morrison, 2021). Planning ahead, allocating sufficient resources and time to morphotyping, and obtaining accurate taxonomic identifications are thus investments that improve data quality and the long‐term value of ecological datasets.

## Rule 2: Keep permanent records to verify identifications

While some taxa can be reliably identified in the field, identifications should be associated with permanent records. Voucher specimens (Box 1) stored in physical and/or digital form in accessible biological collections are ideal because they provide verifiable evidence (Culley, 2013; Baker *et al*., 2017; Davis, 2023). Theoretically, all individuals should be identified using voucher specimens, particularly in species‐rich communities where misidentification is more likely and/or where species‐level taxonomy is not well established. However, specimen collection and preparation are difficult for some groups (e.g. palms, cacti) or not allowed for protected species (e.g. orchids). In some ecosystems (e.g. grasslands) and large‐scale projects (e.g. national forest inventories), it is unfeasible to sample and permanently store one specimen per individual. In such cases, having at least one specimen per morphotype per site is a practical alternative. Additionally, recording detailed field notes (see Rule 10), storing image vouchers, and/or tissue samples can facilitate verification of identifications. Image vouchers are increasingly complementing physical specimens (Gómez‐Bellver *et al*., 2019), particularly through the emergence of mobile platforms (e.g. iNaturalist and Pl@ntNet) that store images and support community‐based species identification and verification (Campbell *et al*., 2023; Greene *et al*., 2023). Since taxonomy changes over time, records must be stored for future reference, so identifications can be reassessed in the light of new knowledge and technologies (e.g. DNA barcoding or near‐infrared spectroscopy of plant leaves) (Durgante *et al*., 2013; Kuzmina *et al*., 2017; Gaem *et al*., 2022). AI‐assisted plant identification based on images is advancing rapidly and supports the identification of many species (Truong & Van der Wal, 2024; Antonelli *et al*., 2026). However, AI‐assisted identification remains constrained by a lack of taxonomist‐verified training data, particularly in species‐rich tropical floras, reinforcing the need for systematically storing image vouchers.

## Rule 3: Keep records of who did the identifications

The ability to identify species varies between people, and even the same person may provide different identifications of the same species over time (Gomes *et al*., 2013). Trained taxonomists are the primary authority for accurate species identification, but access to such expertise may be limited for different reasons (Baker *et al*., 2017; Engel *et al*., 2021). Some ecological studies rely on parataxonomists (Box 1) who can sort organisms into morphotypes (Box 1) and provide valuable identifications, which are effective for many applications. In addition, AI‐based tools are increasingly being used to assist species identification. However, identifications not made by taxonomists or AI‐assisted identification based on inadequate training data may lack the precision required for many species and for purposes such as conservation planning or studying beta‐diversity patterns (Krell, 2004; Procópio & Secco, 2008). Given the importance of species identifications for downstream analyses, it is important to document who identified each individual organism, where and when, so that confidence levels can be assigned, even if the level of taxonomic training is not always a good proxy for confidence in identification across all taxa (Gomes *et al*., 2013). See Rule 10 for other important field information that should be recorded to support species identification and the subsequent use and interpretation of the data.

## Rule 4: Follow a taxonomic backbone

A taxonomic backbone is an authoritative list of taxon names, their authors, and status (i.e. as accepted names, synonyms, or orthographic variants). Using a single backbone provides a consistent framework that allows names to be compared across surveys and ensures the integrity of downstream analyses. For vascular plants, there are global‐scale backbones (Costa *et al*., 2023) such as the World Flora Online (Borsch *et al*., 2020). Taxonomic backbones vary in their content and completeness, how they are compiled (e.g. by individuals or large community consortia), and how often they are updated (Grenié *et al*., 2023). This means that the most appropriate backbone should be selected for each study group and geographical region. Additionally, as backbones can be continuously updated through taxonomic revisions, the version used should also be reported. If a backbone is not followed, it is important to ensure that the taxon names and authors have been published. For plants, all such names should be in the International Plant Names Index.

## Rule 5: Store full taxonomic information

Although often seen as a detail, it is important to store full scientific names (i.e. Latin binomial names and their full authors) to avoid confusion caused by synonyms (i.e. different names for the same species; see Box 1). Synonyms can be ‘heterotypic’ (i.e. when different species names are based on different type specimens) or ‘homotypic’ (i.e. when synonyms are based on the same type specimen, with frequent examples when the limits of genera are redefined). Much rarer, but more problematic, are ‘homonyms’ (i.e. species with the same name described by different authors, based on different type specimens, which actually belong to different species). Therefore, having full scientific names with their authors is key to accurate cross‐site comparisons and cross‐region data integration. Whenever available or possible, infra‐specific names (e.g. varieties, subspecies) should also be stored, independently of the taxonomic level at which subsequent analyses will be carried out. Infra‐specific names carry important population information and can also help to keep the dataset up to date with future taxonomic revisions and circumscriptions (Borges *et al*., 2017; Damasco *et al*., 2021) (also see Box 2). It is also important to store the rank of the taxon name (e.g. genus, species, subspecies) and to avoid confusion between accepted species names and the informal or field names applied to morphotypes (Box 1 and Rule 7). For extensive datasets, lists in accessible formats containing the full taxonomic information can be provided as digital appendices or deposited in online repositories, fostering data transparency, verification, and reuse.

## Rule 6: Use correct taxonomic terminology

Taxonomy has its own terms and conventions ruled by codes such as the International Code of Nomenclature for algae, fungi, and plants (Turland *et al*., 2025), which define taxonomic terminology and when to use it. Adhering to these codes improves consistency within ecological datasets and facilitates communication across disciplines. For instance, there are conceptual and applied differences between the taxon name qualifiers ‘cf.’ (*confer*) and ‘aff.’ (*affinis*), and between ‘sp.’ (species) and ‘indet.’ (*species indeterminabilis*), which should be understood to ensure their correct use (Sigovini *et al*., 2016). Ideally, such name qualifiers should be stored in a dedicated dataset field to ease downstream name standardization and matching (see Rule 9). Taxonomic terms can be provided in full or abbreviated, so it is important to be consistent in choosing between options and to follow the format already adopted by biological or ecological data standards as closely as possible (see Rule 10).

## Rule 7: Deal properly with incomplete identifications

Organisms often cannot be identified to a taxonomically recognized species in ecological surveys, but morphologically similar individuals can still be grouped into a single putative species. These entities are commonly called ‘morphotypes’ or ‘morphospecies’ and are critical for many ecological analyses (e.g. for understanding alpha‐diversity). That is, they are not merely placeholders for incomplete identifications, but operational taxonomic units, hypothesized to represent individual species. Therefore, besides the use of reliable protocols for field morphotyping (Rule 1), they should be explicitly linked to images or voucher specimens (Rule 2), particularly in species‐rich tropical forests and in projects involving multiple people performing morphotyping. Morphotypes should also be explicitly indicated in a dataset field, such as the one containing the taxonomic ranks of the individuals or in a separate field. Sequential and unique identifiers are recommended, for example, Genus sp.1 (e.g. *Andira* sp.1), Family sp.1 (e.g. Fabaceae sp.1), and Unidentified sp.1. It is helpful to use a consistent format for the identifiers throughout the dataset (e.g. use ‘sp.1’ OR ‘sp. 1’). Informal field names for morphotypes that can be easily confused with existing species names should be avoided, even if they have meanings that can help morphotyping individuals (e.g. ‘pilosa’, ‘glabra’, ‘discolor’, ‘hirsuta’, etc.). If these field names cannot be avoided, they should be stored in a separate field rather than embedded in the one containing scientific names, since they can create erroneous name matches while using automated name‐matching tools (Rule 9).

## Rule 8: Keep track of identification changes

Taxonomic revisions can lead to changes in species boundaries, with previously recognized species being merged or a single species being split into multiple species, sometimes by elevating infra‐specific ranks to species level (Borges *et al*., 2017; Damasco *et al*., 2021). So, although checking taxon names against a backbone is advised (Rule 4), both the original identifications (i.e. names as first identified or provided), and any subsequent name changes should also be stored. This additional information allows the updating of the taxonomy (e.g. synonyms or orthography) without losing track of those changes, and it also allows safe switching between backbones (Richard‐Bollans *et al*., 2026). If original names were corrected for synonyms or typographical errors (see Rule 9), the history of name changes should be recorded within the dataset itself (e.g. original, corrected, and accepted names). Moreover, it is not uncommon to find synonyms and their accepted names in the same sample of an ecological dataset (e.g. an inventory plot). They may belong to the same species, or they may indicate two different morphotypes that were correctly distinguished in the field but not associated with the correct species, risking their lumping during taxonomic standardization. Some cases cannot be solved, but keeping the complete history of name changes, including who identified them and when (Rule 3), facilitates tracking taxonomic updates. Storing dataset copies after major changes in repositories with persistent identifiers (e.g. Zenodo, Dryad) is recommended to ensure reproducibility.

## Rule 9: Use name‐matching tools carefully

Several computational tools are available to match species names to a taxonomic backbone (Grenié *et al*., 2023). These tools are useful for checking for typos and updating your taxonomy (e.g. new synonyms). However, matching names, particularly fuzzy matching, carries risks (Grenié *et al*., 2023). First, automated name matching should rely on a taxonomic backbone with good coverage for the groups of interest, to avoid false positive fuzzy matches (e.g. *Myrcia grandifolia* vs *Myrcia grandiflora*). When calculating name distances, taxonomic ranks (e.g. var., subsp.), name qualifiers (i.e. cf., aff.), and author names should be consistently included or excluded in both input and reference names. Fuzzy matching tools often use fixed proportions of maximum string distance (e.g. Levenshtein distance), which can result in omission or commission depending solely on the length of the input name, particularly for matches including author names. The manual inspection of the output of those tools is recommended to avoid erroneous name matches in the dataset.

Box 1 **Terms and definitions often encountered when working with plant taxonomic data. Terminology was obtained from the International Code of Nomenclature for algae, fungi, and plants (Turland *et al*., 2025) and from Judd *et al*. (2007)**

**Table:** 

Term/Definition	Description
Accepted name	Taxon name that complies with the International Code and reflects current taxonomic consensus.
aff. (*affinis*)	Qualifier indicating that a specimen has an affinity to, but is not identical to, a named taxon, suggesting, for example, the possibility of a new species. For example, *Ocotea* aff. *porosa* suggests a species closely related to, but distinct from, *Ocotea porosa*.
Author names (and abbreviations)	Name of the taxonomist who described and published a taxon. Name abbreviations are standardized to ensure consistency. For example, ‘L.’ represents Carl Linnaeus and only him.
cf. (*confer*)	Qualifier indicating that a specimen likely is a known taxon but requires further verification. For example, the designation *Ocotea* cf. *porosa* indicates that a specimen is similar to *Ocotea porosa*.
ex	The preposition ‘ex’ in an author citation indicates that a name was first proposed by one author but was validly published by a different author (the publishing author).
f. (forma)	An infra‐specific rank that denotes minor morphological variants within a species. Typically used when differences are insufficient to warrant recognition at a higher taxonomic level.
Field name	An informal name used in field surveys to refer to the identity of a morphotype. Formal taxonomic and nomenclatural rules do not govern them.
Fuzzy matching	A computational technique for identifying text matches, such as taxon names, while accounting for typos or spelling variants.
Heterotypic (taxonomic) synonym	A name that refers to the same taxon as an accepted name but is based upon a different type specimen. Treatment as a heterotypic synonym represents the current taxonomic judgment that it and the accepted name represent the same taxon (e.g. species).
Homotypic (nomenclatural) synonym	A taxon name that shares the same type specimen as the accepted name but is no longer in use.
Indet	Abbreviation for ‘indeterminate’, often used when an individual cannot be conclusively identified to a taxon.
Morphospecies or Morphotype	A provisionally delimited but not formally described species defined based on morphological traits.
Orthographic variant	A spelling variant of a taxon name arising from typographical errors or differences in transliteration. It does not indicate any taxonomic difference.
Parataxonomist	A professional, often with extensive field experience but not necessarily formal training in taxonomy, who assists in the collection, sorting and identification of individual organisms.
sp.	An abbreviation indicating that the species epithet is unknown, unspecified, or not determined (e.g. *Ocotea* sp.). See ‘Indet’.
spp.	Abbreviation used to refer to multiple species in the same genus (e.g. *Ocotea* spp.). The plural form of sp. (see above).
Subspecies	An infra‐specific rank that designates an often geographically isolated population with a distinguishable set of attributes.^1^
Synonym	A scientific name assigned to a taxon, but that is no longer the currently accepted name. This term includes both homotypic and heterotypic synonyms (see above).
Type	Designated specimen or illustration that serves as the definitive reference for a taxon name.
Valid publication	The formal publication of a taxon name in accordance with the nomenclatural rules, including a taxon description, designation of a type, in an acceptable medium of dissemination.
Variety	An infra‐specific rank that designates a population or populations that show minor morphological differences within a species. This term is only used for plant species.^1^
Voucher specimen	A preserved specimen deposited in a collection (e.g. herbarium or museum) that serves as verifiable evidence for the occurrence and identification of a taxon.

^1^On the specific cases of ‘subspecies’ and ‘variety’, the code regulates the hierarchy, establishing that a subspecies ranks above a variety. However, it does not provide biological criteria for distinguishing them.

Box 2 **Common mistakes in the management of taxonomic information**

**1. Scientific names without author names**

Scientific names lacking author names can lead to ambiguous name matches (Rule 5). Querying *Artemisia rupestris* in the World Flora Online returns eight possible names published by different authors for different species (Richard‐Bollans *et al*., 2026). For example, *Artemisia rupestris* L. is a subshrub under active pharmacological study, while *Artemisia rupestris* All. is now a synonym of *Artemisia umbelliformis* Lam., the Alpine ‘genepì’ used in Génépi liqueur. So, for a record of *Artemisia rupestris* without the author's name, it is difficult to assign the currently accepted species.

**2. Ignoring infra‐specific names**

Where species boundaries are complex (e.g. in species complexes), infra‐specific names can improve resolution for accurate name‐matching. For example, *Protium heptaphyllum* (Aubl.) Marchand, a hyperdominant species in Amazonia (ter Steege *et al*., 2020), has been recognized as a species complex (Damasco *et al*., 2021). Two taxa within this complex, *Protium cordatum* Huber and *Protium guianense* (Aubl.) Marchand, were originally recognized as separate species, but they were subsequently reduced to infra‐specific rank within *Protium heptaphyllum* (Aubl.) Marchand for decades, as *P*. *heptaphyllum* subsp. *cordatum* (Huber) Daly and *P*. *heptaphyllum* var. *puberulum* Engl., respectively. Recent molecular and morphological evidence (Damasco *et al*., 2021) confirmed that these entities are indeed distinct species with overlapping distributions. Therefore, records omitting prior infra‐specific names would prevent assignment to their correct species as taxonomy evolves (Rule 5).

**3. Failing to preserve voucher specimens and/or images**

Genera with historically complex circumscription, such as *Licania* Aubl., the most species‐rich genus in the Chrysobalanaceae, present significant challenges. This genus was redefined, and eight genera are now recognized (Sothers *et al*., 2016). Consequently, historical records identified to the genus level as *Licania* sp. can no longer be confidently assigned to a current genus without a voucher for confirmation (Rule 2), as these new genera also have overlapping distributions.

**4. Not checking the names before the analyses**

Due to its large distribution range and historical taxonomic revisions, *Myrcia splendens* (Sw.) DC. has 177 synonyms. Any ecological work using this species across its full geographic range should always recognize that multiple synonyms may be present in the data, reinforcing the need to follow the most up‐to‐date taxonomic backbone (Rule 4) and to use name‐matching tools correctly (Rule 9) before analysis.

## Rule 10: Provide rich metadata and follow data standards

Recording comprehensive information associated with organisms is useful for improving species identifications, as well as biogeographic, taxonomic, and ecological knowledge (Baker *et al*., 2017; Miralles *et al*., 2020). For instance, field notes reporting the growth form, habitat, phenological state, associated species (e.g. symbionts) and any other information that is lost or altered during specimen preparation (e.g. size, color, odor) can be important for identification (Sullivan & Nazaire, 2022). Locality information can also help taxonomists relocate individuals in the field if there is any suspicion that a morphotype is a species new to science. It is advisable, therefore, to provide information on the identification and/or position of individuals in the field (e.g. tag numbers in vegetation plots) and on the sampling locality, including geo‐coordinates, elevation, habitat type and condition (e.g. disturbances, successional stage, human impacts), wider environmental conditions (e.g. soil, topography) (Moonlight *et al*., 2021). Such information should also be added to the metadata of the voucher specimens, so that each specimen is permanently and unambiguously linked to the individual, sample, and census event from which it originated. Although presented here in the final rule, metadata collection should begin at the start of fieldwork, since it underlies recommendations made in the previous rules. And while recording detailed notes increases the time and requires additional effort in the field, it enhances the scientific value of the dataset not only for taxonomy but also for gathering evidence of species autecology.

Furthermore, following established data‐sharing standards is recommended for storing data and metadata (Michener, 2015). Although not an ecological standard per se, the Darwin Core standard (DwC; Wieczorek *et al*., 2012) is widely used and offers dedicated fields that accommodate many of the recommendations made above, such as ‘identifiedBy’ for recording who made the identifications (Rule 3) and ‘identificationQualifier’ for storing name qualifiers (Rule 6). The standard also facilitates the storage of information on habitat, field notes, associated taxa, etc. (see full list of fields at https://dwc.tdwg.org/terms). Standards specific to ecological data also exist, such as the Ecological Metadata Language (EML; Fegraus *et al*., 2005), VegX (for vegetation plots; Wiser *et al*., 2011), and AusTraits (for plant traits; Wenk *et al*., 2024), although these are less widely adopted than DwC. But regardless of the standard adopted, even a partial alignment will greatly improve data integration and long‐term reuse.

## Conclusions

Taxonomic information plays a vital role in ecology. The 10 rules presented here, if applied as a general guide, will improve data confidence and quality and lead to better ecological inferences. Although simple in principle, the rules proposed here are not always easy to implement and applying them is time‐ and resource‐consuming. These practical constraints help explain why integrating rigorous taxonomic practices into ecology has been a long‐term, persistent challenge. Davidson (1952) argued decades ago that ecologists need a deeper engagement with taxonomic principles to validate their ecological conclusions. Collaboration with taxonomists, ideally from the early stages of project planning and sampling, helps not only to ensure consistent morphotype delimitation and accurate species identification, but also the correct application of taxonomic nomenclature and formatting. Engaging taxonomists in ecological research also strengthens the generation of the robust and comprehensive scientific knowledge needed to address pressing questions, such as how ecosystems are responding to the ongoing climate and biodiversity crises (Baker *et al*., 2017). This interdisciplinary collaboration also values the essential work of taxonomists, which often goes unrecognized. However, the persistence of taxonomic issues in ecological datasets shows that previous calls have been insufficient to drive systemic change. Broader structural solutions, such as the creation of minimum information checklists for ecology (e.g. MIBBI; Taylor *et al*., 2008) and editorial policies mandating minimum standards for scientific publication, could help translate these calls into widespread practice. Collaboration among taxonomists, ecologists, and computer scientists to develop and refine new tools will help make ecological datasets taxonomically more robust, but ultimately taxonomic knowledge and workforce remain central to ensuring the accuracy of any new technology, including AI‐assisted species identification (de Carvalho *et al*., 2007; Engel *et al*., 2021).

## Competing interests

None declared.

## Disclaimer

The New Phytologist Foundation remains neutral with regard to jurisdictional claims in maps and in any institutional affiliations.
